# Systematic literature review of treatments for management of complications of ischemic central retinal vein occlusion

**DOI:** 10.1186/s12886-016-0282-5

**Published:** 2016-07-11

**Authors:** Steven E. Bradshaw, Smeet Gala, Merena Nanavaty, Anshul Shah, Mkaya Mwamburi, Panos Kefalas

**Affiliations:** Valid Insight®, Kemp House, 152 City Road, London, EC1V 2NX UK; Market Access Solutions, 575 State Route 28, Raritan, NJ 08869 USA; Cell Therapy Catapult, 12th Floor Tower Wing, Guy’s Hospital, Great Maze Pond, London, SE1 9RT UK

## Abstract

**Background:**

To understand the clinical and economic outcomes of treatments for managing complications of ischemic central retinal vein occlusion (iCRVO).

**Methods:**

We conducted a systematic literature review by searching multiple databases and ophthalmology conferences from 2004 to 2015. Studies published in English language and populations of age ≥45 years were included. For clinical endpoints, we defined eligibility criteria as randomized controlled trials, prospective before-and-after study designs, and non-randomized studies reporting on treatments in patients with iCRVO. For economic endpoints, all types of study design except cost-of-illness studies were included. We evaluated the definitions of ischemia, clinical and economic endpoints, and rate of development of complications. Risk of bias was assessed for clinical studies using the Cochrane risk-of-bias tool.

**Results:**

A total of 20 studies (1338 patients) were included. Treatments included anti-vascular endothelial growth factors (anti-VEGFs), steroids, and procedures primarily targeting macular edema and neovascularization. Ischemia was not defined consistently in the included studies. The level of evidence was mostly low. Most treatments did not improve visual acuity significantly. Development of treatment complications ranged from 11 to 57 %. Incremental cost-effectiveness ratios reported for anti-VEGFs and steroids were below the accepted threshold of GB£30,000, but considering such treatments only ameliorate disease symptoms they seem relatively expensive.

**Conclusions:**

There is a lack of evidence for any intervention being effective in iCRVO, especially in the prevention of neovascularisation. iCRVO poses a significant clinical and economic burden. There is a need to standardize the definition of ischemia, and for innovative treatments which can significantly improve visual outcomes and prevent neovascular complications.

## Background

Central retinal vein occlusion (CRVO) is a vascular disorder of the eye and a known cause of significant visual morbidity, including sudden blindness [1]. The global burden of CRVO in adults is estimated to be 2.5 million [2]. The incidence of CRVO increases with age by greater than 10-fold from 40 years of age to 65 years and older [3, 4]. The estimated annual direct cost for managing CRVO in the Medicare population was approximately $1.3 billion in 2006 [5]. In addition, the economic burden of CRVO is significantly higher than for glaucoma. The 1- and 3-year per-patient direct medical costs associated with CRVO are 24 and 15 % higher, respectively, than costs associated with glaucoma [6], despite the prevalence of glaucoma being 24-fold greater than CRVO [7].

The available treatments for iCRVO are used off-label and are directed towards minimizing or delaying the onset of complications associated with CRVO, such as macular edema (ME) and neovascularization (NV) [3]. Complications of NV include neovascular glaucoma (NVG) and vitreous hemorrhage (VH), which can lead to severe visual morbidity and blindness [8].

CRVO has two forms: ischemic and non-ischemic. Non-ischemic CRVO is the milder form of the disease that may resolve on its own or may progress to the ischemic form. Ischemic CRVO (iCRVO) is more severe, resulting in NVG and/or VH. Diagnosis and characterization of the severity of CRVO can be achieved through funduscopy, fluorescein angiography, and optical coherence tomography [9, 10]. Ischemia in CRVO is identified by using various criteria based on findings from these examinations or tests [10]. The exact epidemiology of iCRVO remains unknown; however, one study suggests that iCRVO constitutes about one-fifth of all CRVO cases [11]. Another study estimates 15 % of patients with non-ischemic CRVO progress to iCRVO within 4 months and that 34 % progress within 3 years [8].

More than 90 % of patients suffer from partial or complete vision loss if complications of NVG or iris NV are left untreated [12]. Current management of complications of CRVO include intravitreal anti-vascular endothelial growth factors (anti-VEGFs), intravitreal steroid depots, laser treatments, and a range of surgical interventions [3]. The exact rates of complications in patients with iCRVO receiving these off-label treatments remain unknown and have not been systematically evaluated. There is also a need for a comprehensive systematic review documenting evidence on the full range of treatments for iCRVO, their respective complication rates, and the costs associated with these treatments. The objective of this systematic literature review is to document the clinical outcomes, rates of post-treatment complications associated with interventions, and economic outcomes of treatments used to manage complications of iCRVO.

## Methods

### Search methods for identifying studies

We conducted a systematic review using search strategies with Medical Subject Heading (MeSH) terms for iCRVO and clinical outcomes to identify relevant studies. A similar search was performed for economic outcomes; however, the search strategy was not restricted by ischemia-related terms. We searched PubMed, EMBASE, PsycINFO, Education Resources Information Center (ERIC), Cochrane Database of Systematic Reviews (CDSR), Database of Abstracts of Reviews of Effects (DARE), Cochrane Central Register of Controlled Trials (CENTRAL), Health Technology Assessment (HTA) database, and the Campbell Collaboration Library of Systematic Reviews. In addition to the above literature sources the UK National Health Service (NHS) Economic Evaluation Database (NHS EED) was searched for economic studies. The searches were limited to the period January 2004–March 2015, as guidelines on management of CRVO by the UK Royal College of Ophthalmologists were first published in 2004, and to the English language and human studies. Conference proceedings from EURetina, Royal College of Ophthalmologists (available for 2013 and 2014), American Academy of Ophthalmology (AAO), and International Society of Pharmacoeconomics and Outcomes Research (ISPOR) were also searched for 2004–2015. The search strategy for the PubMed database is shown in Table 1.

**Table 1:** Search strategy for the PubMed database

Search number	Search strategy	Number of hits
1	((“Central Retinal Vein Occlusion”[Title/Abstract]) OR CRVO[Title/Abstract]) OR vein, central retinal[MeSH Terms] Filters: Publication date from 2004/01/01 to 2015/03/01; Humans; English	1058
2	((Central Retinal Vein Occlusion[Title/Abstract]) OR CRVO[Title/Abstract]) OR vein, central retinal[MeSH Terms]) AND (ishaemic[Title/Abstract] OR ischemic[Title/Abstract]) Filters: Publication date from 2004/01/01 to 2015/03/01; Humans; English	127
3	(((“Central Retinal Vein Occlusion”[Title/Abstract]) OR CRVO[Title/Abstract]) OR vein, central retinal[MeSH Terms]) AND (efficacy [Title/Abstract] OR “quality of life” [Title/Abstract] OR effectiv* [Title/Abstract] OR “treatment outcome” [Title/Abstract] OR treatment outcome [MeSH Terms] OR quality of life [MeSH Terms]) Filters: Publication date from 2004/01/01 to 2015/03/01; Humans; English	273
4	(((Central Retinal Vein Occlusion[Title/Abstract]) OR CRVO[Title/Abstract]) OR vein, central retinal[MeSH Terms]) AND (economic [Title/Abstract] OR cost [Title/Abstract] OR “cost analysis” [Title/Abstract] OR cost-effective* [Title/Abstract] OR “treatment cost” [Title/Abstract] OR “health care cost” [Title/Abstract] OR utility [Title/Abstract] OR reimbursement [Title/Abstract] OR “drug cost” [Title/Abstract] OR “cost saving”[Title/Abstract] OR “unit cost” [Title/Abstract] OR Health Expenditures[MeSH Terms] OR Drug Costs[MeSH Terms] OR Cost Sharing[MeSH Terms] OR Cost of Illness[MeSH Terms] OR Cost Savings[MeSH Terms] OR Technology, High-Cost[MeSH Terms] OR Cost Control[MeSH Terms] OR Cost-Benefit Analysis[MeSH Terms] OR Cost Allocation[MeSH Terms] OR Direct Service Costs[MeSH Terms] OR Hospital Costs[MeSH Terms] OR Employer Health Costs[MeSH Terms]) Filters: Publication date from 2004/00/01 to 2015/03/01; Humans; English	8
Final		1466

### Eligibility criteria

We included studies assessing individuals 45 years or older with complications of iCRVO. Study populations were considered ischemic if at least one of the following was present: a) the study mentioned the population had ischemia or non-perfusion and b) the inclusion criteria of the study included at least one of the Hayreh [9] or Central Retinal Vein Occlusion Study (CVOS) [10] criteria. Hayreh’s criteria [9] include: a) presence of multiple dark deep intraretinal hemorrhages, b) presence of multiple cotton wool spots, c) degree of retinal vein dilatation and tortuosity, d) relative afferent pupillary defect, and e) electroretinographic tests showing reduced b-wave amplitude, reduced b:a ratio, and prolonged b-wave implicit time. The CVOS criteria [10] include: a) poor visual acuity of <6/60 (equivalent decimal scale = 0.10 and logarithm of the minimum angle of resolution (LogMAR) = 1.00) and b) fluorescein angiography showing greater than 10 disc areas of retinal capillary non-perfusion. Clinical studies were excluded if the results were not reported separately by ischemic status. If a study reported results by ischemic status then the results for the ischemic subpopulation were included in this review. However, if ischemia was not explicitly mentioned in the economic studies, ischemia was determined manually by authors during the study selection phase. Economic studies with CRVO population having complications such as persistent ME and NV were considered as ischemic, and hence were included.

We included studies of interventions used in clinical practice to manage iCRVO or its complications against any comparator (sham, placebo, other active treatment/intervention). Studies without comparator but reporting before-and-after outcomes were also included. We focused on studies reporting clinical outcomes such as visual acuity and retinal thickness, and/or rates of complication development, prognosis of complications, relationship between complications and economic outcomes such as cost of treatment, cost per quality-adjusted life year (QALY), and incremental cost-effectiveness ratio (ICER).

Randomized controlled trials (RCTs), non-randomized trials, and prospective uncontrolled (before-and-after) study designs were included to assess clinical outcomes. All economic studies except cost-of-illness studies were included. Retrospective studies, case studies, commentaries, and case series were excluded. Systematic reviews and meta-analysis were used to cross-reference bibliographies to ensure relevant studies were not inadvertently excluded.

### Study selection

Abstracts identified by the search were screened independently by two reviewers and any differences were resolved by consulting a third arbitrator.

### Data collection and risk-of-bias assessment

Data from eligible studies were extracted and information was collected for country of investigation, sample size, inclusion and exclusion criteria, patient characteristics at baseline, efficacy outcomes, rate of complication development, relationship between complications, type of economic analysis, perspective of the analysis, cost year, quality of life, and economic outcomes. All best corrected visual acuity (BCVA) values were converted to LogMAR units [13]. All costs were converted to 2015 GBP using the Organisation for Economic Co-operation and Development gross domestic product purchasing power parity conversion rates [14]. Data were extracted by one reviewer and 100 % verified by a second reviewer. Risk of bias for each clinical study was assessed using the Cochrane risk-of-bias assessment tool [15].

## Results

### Study selection

**Fig. 1:**
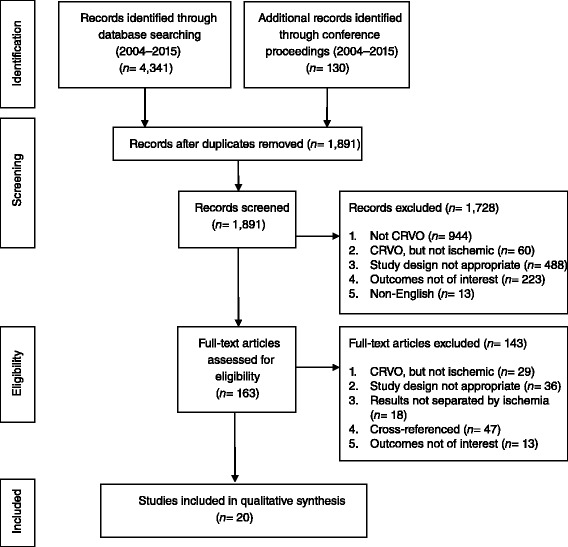
PRISMA 2009 flow diagram. CRVO = central retinal vein occlusion

A total of 1891 de-duplicated study abstracts including 130 conference abstracts were screened, of which 20 studies (13 reporting clinical outcomes and seven reporting economic outcomes) were included in the final assessment. A flow diagram summarizing the study attrition is shown in Fig. 1.

### Study characteristics

In the included 13 clinical studies shown in Table 2, six studies reported ME complications, four studies reported NV complications, and three studies did not mention complications. Seven studies [16–22] were RCTs and two [23, 24] were before-and-after prospective uncontrolled studies. Other prospective study designs included non-RCTs [25, 26], a randomized trial assessing two doses of the same drug [27], and a cohort study [28]. The included studies were conducted in various countries across the world, including Iran [21, 23, 25] (*n* = 3), USA [27, 28] (*n* = 2), Germany [24, 26] (*n* = 2), Sweden [16] (*n* = 1), Italy/USA [22] (*n* = 1), and Japan [20] (*n* = 1). Three were multinational [17–19]. Anti-VEGF treatments included aflibercept, bevacizumab, and ranibizumab; steroid treatments included triamcinolone; procedural treatments included pars plana vitrectomy with radial optic neurotomy, panretinal photocoagulation (PRP), selective PRP, photodynamic therapy with verteporfin, retinal endovascular lysis, and surgical induction of chorioretinal venous anastomosis; aspirin was used as an anticoagulant.

**Table 2:** Study characteristics of the included clinical studies

Study (country)	Study design	Complication secondary to iCRVO	Follow-up period (months)	Treatment arm (% with ischemia in overall CRVO patient population)^l^	Comparator arm (% with ischemia in overall CRVO patient population)^l^	Age in years (treatment vs. comparator)	Proportion of females (treatment vs. comparator)
ANTI-VEGF TREATMENTS							
Korobelnik et al. 2014 [17] (multinational)	Prospective, randomized, double-masked, sham-controlled clinical trial	ME^a^	13	▪ Intravitreal aflibercept injection	▪ Sham injection	▪ NA^k^	▪ NA^k^
				o *n* = 7 (6.8 % non-perfused of 103)	o *n* = 7 (10.3 % non-perfused of 68)		
Brown et al. 2013 [19] (multinational)	Prospective, randomized, double-masked, sham-controlled clinical trial	ME^a^	12	▪ Intravitreal aflibercept injection	▪ Sham injection	▪ NA^k^	▪ NA^k^
				o *n* = 17 (14.9 % non-perfused of 114)	o *n* = 12 (16.4 % non-perfused of 73)		
Boyer et al. 2012 [18] (multinational)	Prospective, randomized, double-masked, sham-controlled clinical trial	ME^b^	6	▪ Intravitreal VEGF Trap-Eye (aflibercept)	▪ Sham injection	▪ NA^k^	▪ NA^k^
				o *n* = 17 (14.9 % non-perfused of 114)	o *n* = 12 (16.4 % non-perfused of 73)		
Wittstrom et al. 2012 [16] (Sweden)	Randomized, clinical pilot study	NVG^c^	6	▪ Single intravitreal injection of bevacizumab combined with PRP	▪ PRP	▪ 78.4 (7.8) vs. 78.0 (8.7)	▪ 80 % vs. 44 %
				o *n* = 9 (100 % ischemic)	o *n* = 9 (100 % ischemic)		
Campochiaro et al. 2008 [27] (USA)	Prospective, randomized, uncontrolled open-label, double-masked trial	ME^d^	3	▪ Ranibizumab 0.3 mg (3-monthly injections)	▪ Ranibizumab 0.5 mg (3-monthly injections)	▪ 63 (17) vs. 68 (13)	▪ NA
				o *n* = 10 (100 % ischemic)	o *n* = 10 (100 % ischemic)		
STEROID TREATMENTS							
Asano et al. 2007 [20](Japan)	Randomized controlled study	Ischemic CME or ME^e^	4 (all eyes underwent laser treatment prior to study)	▪ Sub-tenon triamcinolone injection	▪ No sub-tenon triamcinolone injection	▪ 64.0 (7.1) vs. 65.1 (6.4)	▪ 47 % vs. 47 %
				o *n* = 15 (100 % ischemic)	o *n* = 15 (100 % ischemic)		
Ramezani et al. 2006 [21] (Iran)	Randomized, sham-controlled clinical trial	NV preventive effect^f^; 52 % were ischemic	4	▪ Intravitreal triamcinolone	▪ Sham subconjunctival injection	▪ NA^k^	▪ NA^k^
				o *n* = 9 (69 % non-perfused of 13 eyes)	o *n* = 4 (29 % non-perfused of 14 eyes)		
Jonas et al. 2005 [26] (Germany)	Prospective, non-randomized, clinical interventional study	CME^c^	Treatment: 10.1 (mean); comparator: 6.0 (mean)	▪ Triamcinolone acetonide intravitreal injection (about 20 mg)	▪ No treatment (results were not given by ischemic status)	▪ NA^k^	▪ NA^k^
				o *n* = 4 (31 % ischemic eyes of 13 eyes)	o *n* = 5 (25 % ischemic eyes of 20 eyes)		
PROCEDURAL TREATMENTS							
Tabatabaii et al. 2008 [23] (Iran)	Interventional case series study	Not mentioned^c^	3.6	▪ Pars plana vitrectomy with radial optic neurotomy	▪ Pre-operation	▪ 56	▪ 44 %
				o *n* = 18 eyes of 16 patients (100 % ischemic)			
Parodi et al. 2007 [22] (Italy and USA)	Prospective, randomized clinical trial	Anterior-segment NV^g^	12	▪ Conventional PRP (performed promptly when two clock hours of iris NV, any angle NV, or both were identified)	▪ Arm 1—Selective PRP (performed only in selected cases showing progression of iris NV, angle NV, or both during weekly follow-up)	▪ 69.4 (4.1) vs. 69.5 (5.6) [Arm 1] vs. 67.7 (4.9) [Arm 2]	▪ 42 % vs. 30 % [Arm 1] vs. 39 % [Arm 2]
				o *n* = 19 eyes (100 % ischemic)	o *n* = 20 eyes (100 % ischemic)		
					▪ Arm 2—Photodynamic therapy with verteporfin (directed at the iris NV and angle NV)		
					o *n* = 18 eyes (100 % ischemic)		
Feltgen et al. 2007 [24] (Germany)	Prospective, non-randomized, interventional case series	Not mentioned^h^	12	▪ Retinal endovascular lysis	▪ Pre-operation	▪ 67	▪ NA
				o n =13 (100 % ischemic)			
Mirshahi et al. 2005 [25] (Iran)	Non-randomized controlled trial	Prevention of NV^i^	6–18 (mean = 10)	▪ Surgical induction of chorioretinal venous anastomosis	▪ No surgery	▪ NA	▪ 60 % vs. 39 %
				o *n* = 10 (100 % ischemic)	o *n* = 18 (100 % ischemic)		
MISCELLANEOUS TREATMENTS							
Hayreh et al. 2011 [28] (USA)	Prospective study	Not mentioned^j^	Treatment: 22.8 (median); comparator: 34.8 (median)	▪ Aspirin	▪ No aspirin or anticoagulant	▪ 70 (12) vs. 68 (16)	▪ 42 % vs. 53 %
				o *n* = 38 (17 % ischemic of 227)	o *n* = 47 (15 % ischemic of 324)		

definitions of ischemia used by study

*BCVA* best corrected visual acuity, *CME* cystoid macular edema, *CVOS* Central Retinal Vein Occlusion Study, *iCRVO* ischemic central retinal vein occlusion, *ME* macular edema, *NA* not available, *NV* neovascularization, *NVG* neovascular glaucoma, *PRP* panretinal photocoagulation, *VEGF* vascular endothelial growth factor

^a^≥10 disc areas of non-perfusion (CVOS classification)

^b^BCVA of 20/40 to 20/320 and ≥10 disc areas of non-perfusion

^c^Not reported

^d^BCVA of 20/30 to 20/400

^e^Large non-perfusion areas, severe hemorrhages, and severe dye leakage

^f^Capillary non-perfusion on fluorescein angiography, afferent pupillary defect, visual acuity, severity of intraretinal hemorrhages

^g^Reduced b-wave amplitude on electroretinography and ≥10 disc areas of capillary non-perfusion on fluorescein angiography (CVOS classification)

^h^CVOS classification

^i^BCVA <20/200, the presence of a relative afferent pupillary defect of 2+ or more, extensive retinal hemorrhage, ≥10 disc areas of capillary non-perfusion, and the absence of NV

^j^Hayreh’s classification

^k^Data were not reported for ischemic patients separately

^l^Number of patients reported refers to the population included in the final analysis sets

Cost and economic outcome data were available from seven studies, of which four [29–32] used cost-utility analysis and three [33–35] used cost-effectiveness analysis. Of the seven studies, six [29–32, 34, 35] were obtained from relevant conference proceedings and only one [33] was a full-text article, leading to lack of comprehensiveness in reported data. All studies were conducted in CRVO patients, without the ischemic status provided explicitly. However, NV complications and/or persistent ME are typically associated with ischemia; hence we considered these economic studies relevant to the iCRVO population. Table 3 reports the key characteristics of the studies included.

**Table 3:** Summary of included economic analyses

Study, country	Form of economic analysis	Treatment	Comparator	Patient population	Model horizon
Taylor et al., 2014 [33] UK	Cost-effectiveness	Ranibizumab	Observation	Patients with ME secondary to CRVO	Lifetime
Eriksson et al., 2014 [29] Sweden	Cost-effectiveness	Aflibercept	Ranibizumab	Patients with ME secondary to CRVO; average starting age 64 years	15 years
Duff et al., 2012 [31] USA	Cost-utility	1) Ranibizumab	1) Dexamethasone intravitreal implant	Patients with ME secondary to CRVO	2 years
		2) Dexamethasone intravitreal implant	2) Steroids		
Haig et al., 2012 [32] Canada	Cost-utility	Ranibizumab	Observation	Patients (66–68 years) with ME secondary to CRVO	Lifetime
Vincente et al., 2013 [30] Canada	Cost-effectiveness	Dexamethasone intravitreal implant	Observation	Patients with ME and vision loss secondary to CRVO	Lifetime
Hayward et al., 2011 [34] UK	Cost-utility	Dexamethasone intravitreal implant	Observation	Patients with ME secondary to CRVO from GENEVA 008 and 009 clinical trial studies	Lifetime
Kowalski et al., 2011 [35] USA	Cost-utility	Dexamethasone intravitreal implant	Observation	Individual patient-level data were pooled from phase 3 studies of patients with ME following CRVO; mean age 65 years and study-eye visual acuity of 20/80	Lifetime

*CRVO* central retinal vein occlusion, *ME* macular edema

### Definitions of ischemia

Korobelnik et al. [17], Brown et al. [19], Parodi et al. [22], and Feltgen et al. [24] classified eyes with greater than 10 disc areas of non-perfusion as ischemic; Hayreh et al. [28] used Hayreh’s classification to identify ischemia. Boyer et al. [18] defined ischemia as eyes with a BCVA of 20/40 (+0.3) to 20/320 (+1.2) and greater than 10 disc areas of non-perfusion. Campochiaro et al. [27] considered eyes with a BCVA of 20/30 (+0.2) to 20/400 (+1.3) as ischemic. Asano et al. [20] classified ischemia as eyes having large non-perfusion areas, severe hemorrhages, and severe dye leakage, whereas Ramezani et al. [21] classified ischemia as eyes with capillary non-perfusion, presence of relative afferent pupillary defect (RAPD), poor BCVA, and severe intraretinal hemorrhages. Mirshahi et al. [25] considered a BCVA of less than 20/200 (+1.0), presence of RAPD, extensive hemorrhages, and more than 10 disc areas of non-perfusion as indicating ischemic eyes. Wittstrom et al. [16], Jonas et al. [26], and Tabatabaii et al. [23] did not report their method of classifying ischemia. Definitions of ischemia were not available in the economic studies as none of the studies evaluated costs in ischemic population.

### Clinical outcomes

The commonly reported efficacy and effectiveness endpoints used in studies were BCVA and central retinal thickness (CRT; also referred to as central macular thickness). All studies reported changes in BCVA whereas only six studies [17–21, 27] reported changes in CRT. Among the included studies, BCVA was calculated in different units, such as Snellen visual acuity, LogMAR, and the decimal system.

Studies reported improvement in BCVA and/or reduction in CRT in the treatment group compared to the comparator group; however, most studies did not provide the level of significance of the improvement, and overall the quality of evidence was low mostly owing to the risk of bias and small population sizes (Table 4). BCVA data as reported in studies are shown in Table 5. Data on rate of complication development during or post-treatment were provided by five studies (Table 4) [17, 22–25]. Development of treatment complications ranged from 11 to 57 % [17, 22–25], with NV as the most commonly reported complication during or after treatment. No studies were found that demonstrated a relationship between the different complications of iCRVO.

**Table 4:** Clinical endpoints reported for ischemic central retinal vein occlusion population in the included studies

Study (country)	Complication secondary to iCRVO	Study characteristics	BCVA (SD) converted to LogMAR units (Treatment vs. comparator)	CRT (SD) in μm and/or NV (treatment vs. comparator)	Post-treatment complications (treatment vs. comparator)	Quality of evidence (GRADE)*
ANTI-VEGF TREATMENTS						
Korobelnik et al. 2014 [17] (multinational)	ME	▪ T: Intravitreal aflibercept injection	▪ Mean change in BCVA at: ^a^	▪ Mean reduction in CRT: ^a^	▪ During the 13-month study, NV development: 43 % vs. 43 %	NA
			o 13 months: +17.4 (16.1) vs. −8.0 (15.8)	o 494.6 (318.4) vs. 294.3 (258.6)		
			*BCVA could not be converted to LogMAR units as the baseline BCVA was not available*			
		▪ C: Sham injection				
Brown et al. 2013 [19] (multinational)	ME	▪ T: Intravitreal aflibercept injection	▪ Proportion of eyes gaining ≥ 15 letters at:	Not given by ischemic status	▪ Not given by ischemic status	NA
			o 6 months: 51.4 % vs. 4.3 %			
			o 13 months: 48.6 % vs. 30.4 %			
		▪ C: Sham injection				
Boyer et al. 2012 [18] (multinational)	ME	▪ T: Intravitreal VEGF Trap-Eye (aflibercept)	▪ Mean change in BCVA at: ^a^	▪ Mean reduction in CRT from baseline to: ^b^	▪ Not given by ischemic status	NA
			o 6 months: +17.8 vs. −2.3			
			*BCVA could not be converted to LogMAR units as the baseline was BCVA was not available*	o 6 months: 473.0 vs.309.4		
		▪ C: Sham injection				
			▪ Proportion of eyes gaining ≥ 15 letters at:			
			o 6 months: 51.4 % vs. 4.3 %			
Wittstrom et al. 2012 [16] (Sweden)	NVG	▪ T: Single intravitreal injection of bevacizumab combined with PRP	▪ Mean baseline BCVA:	▪ Median iris NV grade (range):	NA	⊕ ⊕ ⊝⊝ low
			o 1.8 (0.61) vs. 2.0 (0.43)	o At baseline: 2 (0–4) vs. 1 (0–4)		
			▪ Mean BCVA at:			
			o 1 week: 1.8 (0.59) vs. 2.2 (0.45) [*p* = 0.079]	o 1 week: 0 (0–1) vs. 0 (0–3)		
				o 2 months: 0 (0–1) vs. 0 (0–2)		
			o 2 months: 1.8 (0.65) vs. 2.3 (0.46) [*p* = 0.136]	o 6 months: 0 (0–0) vs.0 (0–1) [for T: *p* = 0.001, for C: *p* = 0.005]		
		▪ C: PRP	o 6 months: 1.7 (0.71) vs. 2.3 (0.55) [*p* = 0.114]			
				▪ Median angle NV grade (range):		
				o At baseline: 1.5 (0–4) vs. 1 (0–4)		
				o At 1 week: 1 (0–3) vs. 0 (0–3)		
				o At 2 months: 0 (0–3) vs. 0 (0–3)		
				o At 6 months: 0 (0–3) vs.0 (0–3) [for T: *p* = 0.001, for C: *p* = 0.014]		
Campochiaro et al. 2008 [27] (USA)	ME	▪ T: Ranibizumab 0.3 mg (3-monthly injections)	▪ Mean baseline BCVA:	▪ Mean baseline CRT:	NA	⊕ ⊕ ⊕⊝ moderate
			o 0.78 (1.04–0.07) vs. 0.64 (0.96–0.34)	o 346 (88) vs. 297 (126)		
			▪ Mean BCVA at:	▪ Mean CRT at:		
			o 3 months: 0.44 vs. 0.56	o 3 months: 25 vs. 35 (eliminating 93 % vs. 89 % of the edema, respectively)		
		▪ C: Ranibizumab 0.5 mg (3 monthly injections)				
STEROID TREATMENTS						
Asano et al. 2007 [20] (Japan)	Ischemic CME or ME	▪ T: Sub-tenon triamcinolone injection	▪ Mean baseline BCVA:	▪ Mean baseline CRT:	NA	⊕ ⊕ ⊝⊝ low
			o 2 weeks before injection: 0.501 (0.229) vs. 0.510 (0.141)			
		▪ C: No sub-tenon triamcinolone injection	▪ Mean BCVA at:	o At 2 weeks before injection: 439 (148) vs. 436 (133)		
			o 1 month: 0.463 (0.359) vs. 0.510 (0.169)	▪ Mean CRT at:		
			o 2 months: 0.488 (0.262) vs. 0.501 (0.330)	o 1 month: 315 (142) vs. 443 (150)		
			o 3 months: 0.499 (0.296) vs. 0.501 (0.212)			
			o 4 months: 0.510 (0.203) vs. 0.511 (0.289)	o 2 months: 442 (143) vs. 467 (152)		
				o 3 months: 457 (123) vs. 466 (139)		
				o 4 months: 449 (150) vs. 459 (128)		
Ramezani et al. 2006 [21] (Iran)	NV preventive effect	▪ T: Intravitreal triamcinolone	▪ Mean change in BCVA from baseline to:	▪ Mean change in CRT from baseline to:	Not given by ischemic status	⊕⊝⊝⊝ very low
			o 1 month: −0.40 (0.17) vs. −0.00 (0.12)			
				o 2 months: −273 (108) vs. −115 (71)		
		▪ C: Sham subconjunctival injection				
Jonas et al. 2005 [26] (Germany)	CME	▪ T: Triamcinolone acetonide intravitreal injection (about 20 mg)	▪ Mean baseline BCVA of ischemic patients in treatment arm:	NA	NA	NA
			o 1.79 (0.51)			
			▪ Mean change in BCVA of ischemic patients in treatment arm:			
		▪ C: No treatment (results were not given by ischemic status)	o From baseline to best post-operative VA: 1.57 (0.64) [*p* = 0.10]			
PROCEDURAL TREATMENTS						
Tabatabaii et al. 2008 [23] (Iran)	ME, none of the eyes had NV	▪ T: Pars plana vitrectomy with RON	▪ Mean BCVA:	NA	Post-operation	NA
					▪ Iris NV, VH, and increased intraocular pressure in the early post-operative period: 11 %	
			o Post-operation at 3.6 months vs. pre-operation: 1.32 (0.4–1.9) vs. 1.75 (1.5–1.9) [*p* < 0.01]			
		▪ C: Pre-operation				
					▪ Complicated by retinal detachment requiring pars plana vitrectomy and silicone injection: 5.5 %	
					▪ Development of NV and VH that needed reoperation: 11 %	
Parodi et al. 2007 [22] (Italy and USA)	Anterior-segment NV	▪ T: Conventional PRP (performed promptly when two clock hours of iris NV, any angle NV, or both were identified)	▪ Mean baseline BCVA:	▪ Iris NV (clock hours):	▪ At follow-up, rate of NVG development: 5 % in T and C1 groups, and 11 % in C2 [*p* = 0.713]	⊕ ⊕ ⊕ ⊕ high
			o 1.18 (0.16) vs. 1.19 (0.18) vs. 1.18 (0.15)	o At baseline:3.26 (1.96) [T] vs. 2.95(1.90)[C1] vs. 3.50 (1.97) [C2]		
			▪ Mean BCVA at:			
			o 12 months: 1.23 (0.15) [T] vs. 1.20 (0.18) [C1] vs. 1.15 (0.16) [C2] [*p* = 0.28]			
				o 1 month: 1.05 (1.12) vs. 3.15 (2.08) vs. 0.27 (0.46)		
		▪ C1: Selective PRP (performed only in selected cases showing progression of iris NV, angle NV, or both during weekly follow-up)		o 6 months: 0.47 (1.07) vs. 3.05 (2.21) vs. 1.77 (1.11)		
				o 12 months: 0.52 (2.29) vs. 2.55 (3.05) vs. 2.27 (2.37)		
		▪ C2: Photodynamic therapy with verteporfin (directed at the iris NV and angle NV)				
				▪ Angle NV (clock hours):		
				o At baseline:1.94 (1.12) [T] vs. 1.85 (1.34) [C1] vs. 2.38 (1.88) [C2]		
				o 1 month: 0.68 (0.88) vs. 2.15 (1.81) vs. 0.00 (0.00)		
				o 6 months: 0.52 (1.64) vs. 2.15 (1.89) vs. 0.83 (1.24)		
				o 12 months: 0.57 (2.52) vs. 1.50 (2.64) vs. 1.27 (2.49)		
Feltgen et al. 2007 [24] (Germany)	Not mentioned	▪ T: Retinal endovascular lysis	▪ Mean pre-operative BCVA immediately before surgery:	NA	▪ Rate of post-operative complication development:	NA
		▪ C: Pre-operation	o +1.2 (SEM +1.6/ minus +1.745) (range, +2.6 to +0.70) (N.B: +2.6 = light perception)			
					o NV: 46 %	
					o Retinal detachment: 23 %	
			▪ Mean post-operative BCVA at:			
					o Cataract: 31 %	
					▪ Rate of intra-operative complications:	
			o 6 weeks: +1.31 (SEM plus +1.62/ minus +1.80) (range, +2.6 to +0.4)			
					o Serious retinal detachment: 8 %	
			o 3 months: +1.37 (SEM plus +1.72/ minus +1.85) (range, +2.6 to +0.52)		o VH: 31 %	
					▪ To treat these complications, 13 eyes required 22 additional procedures	
			o 6 months: +1.46 (SEM plus +1.66/ minus +1.89) (range, +2.9 to +0.4) (N.B: +2.9 = Blindness)			
			o 12 months: +1.40 (SEM plus 1.58/ minus +1.80) (range, +2.9 to +0.4)			
			[None of these differences were significant]			
Mirshahi et al. 2005 [25] (Iran)	Prevention of NV	▪ T: Surgical induction of chorioretinal venous anastomosis	▪ Mean BCVA at time period from the onset of occlusion to the time of referral:	NA	▪ Three (30 %) of 10 patients in the treatment arm needed further operations for:	NA
			o 2.5 vs. 1.5 [*p* < 0.001]			
		▪ C: No surgery	▪ Change in BCVA after the occurrence of occlusion:		o Cataract: 33.3 %	
					o Vitreous cavity hemorrhage: 33.3 %	
			o 8 months: gained 0.94 vs. lost 0.57 [*p* < 0.001]			
					o Retinal detachment: 33.3 %	
					o NV: 0 %	
					▪ In the control group, 7 (39 %) of 18 patients developed NV:	
					o NVG: 57 %	
					o Disc NV: 29 %	
					o Iris NV: 14 %	
MISCELLANEOUS TREATMENTS						
Hayreh et al. 2011 [28] (USA)	Not mentioned	▪ T: Aspirin	▪ Baseline BCVA (*n* = 38 vs. 45 eyes) (*p* = 0.905)	NA	NA	NA
		▪ C: No aspirin or anticoagulant				
			o Better than 0.5: 0 (0 %) vs. 0 (0 %)			
			o 0.5–0.7: 1 (3 %) vs. 0 (0 %)			
			o 1.0–1.3: 7 (18 %) vs. 11 (24 %)			
			o CF or worse: 30 (79 %) vs. 34 (76 %)			
			▪ Improved BCVA −0.5 or worse at:			
			o 3 months (*n* = 26 vs. 32): 2 (8 %) vs. 4 (12 %)			
			o 6 months (*n* = 22 vs. 29): 3 (14 %) vs. 5 (17 %)			
			o 9 months (*n* = 18 vs. 32): 3 (17 %) vs. 5 (16 %)			
			o 15 months (*n* = 16 vs. 21): 5 (31 %) vs. 5 (24 %)			
			o 2–5 years (*n* = 9 vs. 17): 2 (22 %) vs. 4 (24 %)			
			▪ Worsened BCVA −0.5 or worse at:			
			o 3 months (*n* = 26 vs. 32): 5 (19 %) vs. 4 (12 %)			
			o 6 months (*n* = 22 vs. 29):4 (18 %) vs. 6 (21 %)			
			o 9 months (*n* = 18 vs. 32): 2 (11 %) vs. 8 (25 %)			
			o 15 months (*n* = 16 vs. 21): 4 (25 %) vs. 5 (24 %)			
			o 2–5 years (*n* = 9 vs. 17): 3 (33 %) vs. 5 (29 %)			

*BCVA* best corrected visual acuity, *C* comparator, *CME* cystoid macular edema, *CRT* central retinal thickness, *iCRVO* ischemic central retinal vein occlusion, *LogMAR* logarithm of the minimum angle of resolution, *ME* macular edema, *NA* not available/not applicable, *NV* neovascularization, *NVG* neovascular glaucoma, *PRP* panretinal photocoagulation, *RON* radial optic neurotomy, *SD* standard deviation, *SEM* standard error mean, *T* treatment, *VEGF* vascular endothelial growth factor

^a^Baseline data were not reported for ischemic patients separately

^b^Quality of evidence were assessed using GRADEpro version 3.6. In order to provide overall consistency in grading methods across studies of primary interest, only prospective, randomized, controlled / uncontrolled clinical trial studies were assessed [45]

**Table 5:** Additional data on the included studies for clinical outcomes

Study (country)	Inclusion criteria, exclusion criteria, baseline co-morbidities	BCVA as reported in the study	Miscellaneous outcomes (treatment vs. comparator)
ANTI-VEGF TREATMENTS			
Korobelnik et al. 2014 [17] (multinational)	▪ Inclusion criteria:	Given in LogMAR (see Table 3)	None
	- Patients had a >50 μm increase in CRT compared with the lowest previous measurement, new or persistent cystic changes within the neurosensory retina or subretinal fluid		
	- Persistent diffuse edema ≥250 μm in the central subfield		
	- Loss of ≥5 letters from the best prior measurement in conjunction with any increase in CRT, or an increase of ≥5 letters in BCVA from the most recent visit, suggesting potentially further improvements upon a subsequent injection		
Brown et al. 2013 [19] (multinational)	▪ Inclusion criteria:	Given in LogMAR (see Table 3)	None
	- Patients aged >18 years		
	- Center-involved ME secondary to CRVO diagnosed within 9 months of study initiation		
	- All study eyes had mean central subfield retinal thickness >250 mm using OCT from Zeiss Stratus OCT (Version 4.0 or later; Carl Zeiss Meditec, Jena, Germany)		
	- Protocol refracted ETDRS12 BCVA of 20/40 to 20/320 (73 to 24 letters)		
	▪ Exclusion criteria:		
	- Any previous treatment with anti-angiogenic drugs; prior panretinal or macular laser photocoagulation; and any ocular disorders that could confound interpretation of study results		
	- Previous use of intraocular corticosteroids or use of periocular corticosteroids within the 3 months prior to day 1		
	- Iris NV, VH, traction retinal detachment, or preretinal fibrosis involving the macula; history or presence of AMD (dry or wet form) that significantly affected central vision; diabetic ME or diabetic retinopathy, defined as eyes of diabetic subjects with more than 1 microaneurysm outside the area of the vein occlusion; and infectious blepharitis, keratitis, scleritis, or conjunctivitis		
Boyer et al. 2012 [18] (multinational)	▪ Inclusion criteria:	Given in LogMAR (see Table 3)	None
	- Patients with eyes whose mean central subfield retinal thickness was 250 μm or more on OCT from Zeiss Stratus OCT		
	- ETDRS BCVA of 20/40 to 20/320 (73 to 24 letters)		
	▪ Exclusion criteria:		
	- Patients with a history of vitreoretinal surgery in the study eye, including RON or sheathotomy, current bilateral retinal vein occlusion, previous panretinal or macular laser photocoagulation		
	- Other causes for decreased VA, ocular conditions with poorer prognosis in the fellow eye		
	- History or presence of AMD, diabetic ME, or diabetic retinopathy, any use of intraocular or periocular corticosteroids, or anti-angiogenic treatment in the study eye at any time or in the fellow eye in the preceding 3 months		
	- Iris NV, VH, traction retinal detachment, or preretinal fibrosis involving the macula, vitreomacular traction or epiretinal membrane that significantly affected central vision, ocular inflammation, uveitis, any intraocular surgery in the preceding 3 months		
	- Aphakia, uncontrolled glaucoma, hypertension, or diabetes, spherical equivalent of a refractive error of more than 8 diopters, myopia, infectious blepharitis, keratitis, scleritis, or conjunctivitis, cerebral vascular accident, or myocardial infarction in the preceding 6 months		
	- Other conditions that may interfere with interpretation of the results or increase the risk of complications		
Wittstrom et al. 2012 [16] (Sweden)	▪ Inclusion criteria:	Given in LogMAR (see Table 3)	▪ Intraocular pressure (mmHg):
	- Patients with iris or anterior chamber angle NV and IOP greater than 22 mmHg were defined as having NVG		○ At baseline: 38.1 (11.1) vs. 38.1 (11.1)
			○ At 1 week: 30.3 (6.6) vs. 24 (11)
	- Open angle was defined as normal angle structures being visible for more than 90°		
			○ At 2 months: 25.2 (8.3) vs. 25.7 (12.4)
			○ At 6 months: 24.8 (12.3) vs. 18.4 (6.8)
	- A closed angle was defined as the presence of peripheral anterior synechiae for more than 270°		
			▪ Rod response (b-wave amplitude):
			○ At baseline: 24.1 (23.0) vs. 22.8 (43.9)
	▪ Exclusion criteria:		
			○ At 6 months: 24.3 (20.5) vs. 17.2 (25.1)
	- Patients with a VA less than light perception, diabetes mellitus, ocular inflammation, or cloudy media due to cataract, keratopathy, VH, a history of thromboembolic disorders including myocardial infarction or cerebrovascular accident and uncontrolled systemic hypertension		
			▪ Rod response (b-wave implicit time):
			○ At baseline: 82.4 (33.0) vs. 56.6 (42.2)
			○ At 6 months: 92.6 (29.4) vs. 72.5 (40.0)
			▪ Standard combined rod/cone response (a-wave amplitude):
			○ At baseline: 45.5 (23.7) vs. 23.8 (20.3)
			○ At 6 months: 18.5 (9.0) vs. 29.2 (21.9)
			▪ Standard combined rod/cone response (a-wave implicit time):
			○ At baseline: 29.7 (5.6) vs. 34.3 (6.9)
			○ At 6 months: 28.7 (5.0) vs. 31.6 (4.9)
			▪ 30-Hz flicker cone(b-wave amplitude):
			○ At baseline: 19.0 (12.4) vs. 14.4 (11.7)
			○ At 6 months: 10.0 (5.2) vs. 12.1 (9.7)
			▪ 30 Hz flicker cone (b-wave implicit time):
			○ At baseline: 42.3 (1.1) vs. 43.6 (2.5)
			○ At 6 months: 42.1 (3.1) vs. 43.8 (1.9)
Campochiaro et al. 2008 [27] (USA)	▪ Inclusion criteria:	▪ Mean baseline BCVA (ETDRS letters at 4 months):	▪ Proportion of patients gaining at least 15 letters (%):
	- Patients >18 years with VA between 20/30 and 20/400 from ME due to CRVO and foveal thickness (central subfield) >250 μm	○ 16 (13) vs. 23 (15)	○ At 3 months: 70 vs. 40
		▪ Mean change in BCVA from baseline to (ETDRS letters at 4 months):	
		○ 3 months: 17 vs. 14	
	▪ Exclusion criteria:		
	- Patients with VA <20/400 in the fellow eye		
	- A sign of possible permanent vision loss in the study eye such as atrophy or prominent pigmentary change in the macula		
	- Laser photocoagulation or intraocular surgery within the previous 3 months		
	- Intraocular injection of a VEGF antagonist within the previous 3 months		
	- Intraocular steroids within the previous 4 months		
	- Vitreomacular traction or an epiretinal membrane		
	▪ Baseline comorbidities: hypertension (55 %), diabetes mellitus (30 %), hyperlipidemia (55 %), elevated homocysteine (20 %), glaucoma (2 %)		
STEROID TREATMENTS			
Asano et al. 2007 [20] (Japan)	▪ Inclusion criteria: not reported	Given in LogMAR (see Table 3)	▪ Mean affected eye/ fellow eye ratio of aqueous flare:
			○ 2 weeks before injection: 2.82 (0.28) vs. 2.77 (0.36)
	▪ Exclusion criteria:		
	- Patients with bilaterally affected eyes		○ 1 month: 1.49 (0.32) vs. 2.64 (0.61) [*p* < 0.05]
	- Those younger than 50 years of age		○ 2 months: 2.38 (0.22) vs. 2.77 (0.28)
	- Eye pathologies related to blood–aqueous barrier breakdown or CME such as diabetes mellitus, uveitis, or previous intraocular surgery		○ 3 months: 2.67 (0.33) vs. 2.75 (0.31)
			○ 4 months: 2.76 (0.28) vs. 2.79 (0.33)
	- Pathologies that could lead to artifacts in aqueous flaremetry, such as advanced cataract or poor mydriasis or allergy to fluorescein sodium		
	- Symptoms for more than 3 months and those that had no detectable ME		
Ramezani et al. 2006 [21] (Iran)	▪ Inclusion criteria:	Given in LogMAR (see Table 3)	None
	- Patients with eyes suffering from iCRVO of less than 2 months’ duration		
	▪ Exclusion criteria:		
	- Patients with monocularity, previous intraocular surgery or laser therapy, VA ≥20/40, glaucoma or ocular hypertension, significant media opacity, NV, accompanying arterial occlusion, signs of chronicity (such as cilioretinal and/or retinal shunt vessels)		
	- Existence of other significant retinal disease		
	- Noncompliance		
Jonas et al. 2005 [26] (Germany)	▪ Inclusion criteria: Not reported	Given in LogMAR (see Table 3)	None
	▪ Exclusion criteria: Not reported		
PROCEDURAL TREATMENTS			
Tabatabaii et al. 2008 [23] (Iran)	▪ Inclusion criteria:	▪ Mean BCVA:	None
	- Patients with onset of CRVO less than 12 months, severe hemorrhage in funduscopy and initial VA worse than 20/400	○ Post-operation at 3.6 months vs. pre-operation: 20/400 (20/1600–20/50) vs. 20/1000 (20/1600–20/630) [*p* < 0.01]	
	▪ Exclusion criteria:		
	- Patients with presence of optic atrophy or macular scar		
	▪ Baseline comorbidities: systemic hypertension (33 %), diabetes mellitus (39 %), open-angle glaucoma (28 %), afferent pupillary defect (83 %)		
Parodi et al. 2007 [22] (Italy and USA)	▪ Inclusion criteria:	Given in LogMAR (see Table 3)	None
	- Patients with diagnosis of iCRVO		
	- presence of two clock hours of iris NV, any angle NV, or both, and availability to undergo both treatment and control examinations		
	▪ Exclusion criteria:		
	- Patients with active hepatitis or clinically significant liver disease, porphyria, or other porphyrin sensitivity		
	- Any previous surgical or laser eye treatment within the past 2 years		
	▪ Baseline comorbidities: hypertension (82 %), cardiovascular disorders (46 %), diabetes mellitus (72 %)		
Feltgen et al. 2007 [24] (Germany)	▪ Inclusion criteria:	▪ Mean post-operative BCVA at:	None
	- Patients with clinically and angiographically diagnosed iCRVO between 6 and 20 weeks after CRVO onset, optimally corrected VA of 0.7 the minimum angle of resolution (LogMAR) or more (decimal VA ≤ 0.2), over 18 years in age	○ 6 weeks: 0.049 + 0.024/ 0.016 (range, LP–0.4)	
		○ 3 months: 0.043 + 0.019/ 0.014 (range, LP–0.3)	
		○ 6 months: 0.035 + 0.022/ 0.013 (range, blindness– 0.4)	
		12 months: 0.04 + 0.026/ 0.016 (range, blindness– 0.4) *[None of these differences were significant]*	
	- Ability to give informed consent		
	▪ Exclusion criteria:	▪ Mean pre-operative BCVA immediately before surgery:	
	- Patients with retinal or disc NV needing photocoagulation at first presentation		
		○ 0.063 + 0.025/ 0.018 (range, LP–0.2)	
	- Other eye diseases that reduced VA, except cataract, e.g., glaucoma with visual-field loss in the other eye		
	- Diabetic retinopathy, macular degeneration, uveitis, vitreous opacity, history of retinal detachment with visual impairment, of retinal vein or artery occlusion, and of neuro-ophthalmological diseases with visual-field defects, amblyopia in the affected eye		
	- Inability to give informed consent		
Mirshahi et al. 2005 [25] (Iran)	▪ Inclusion criteria:	Given in LogMAR (see Table 3)	None
	- Patients with a VA of ≤20/200, the presence of a RAPD pupillary defect of 2+ or more		
	- Extensive retinal hemorrhage		
	- 10 or more disc areas of capillary non-perfusion		
	- Absence of NV		
	▪ Baseline comorbidities: hypercholesterolemia in treatment vs. comparator arms was 60 % vs. 17 %, respectively		
MISCELLANEOUS TREATMENTS			
Hayreh et al. 2011 [28] (USA)	▪ Inclusion criteria:	▪ Baseline BCVA (*n* = 38 vs. 45 eyes) (*p* = 0.905)	None
	- Patients with a definite diagnosis of CRVO	○ Better than 20/70, *n*(%): 0 (0 %) vs. 0 (0 %)	
	▪ Exclusion criteria:	○ 20/70–20/100, *n*(%): 1 (3 %) vs. 0 (0 %)	
	- Patients with all other retinopathies mimicking CRVO or hemi-CRVO		
		○ 20/200–400, *n*(%): 7 (18 %) vs. 11 (24 %)	
	- Inadequate information or doubtful diagnosis, any retinal or optic nerve lesion or any other factor (e.g. cataract), including any treatment for CRVO or hemi-CRVO that could have influenced the visual status		
		○ CF or worse, *n*(%): 30 (79 %) vs. 34 (76 %)	
		▪ Improved BCVA - 20/70 or worse at:	
		○ 3 months (*n* = 26 vs. 32): 2 (8 %) vs. 4 (12 %)	
	- Diagnosis of glaucoma and visual-field loss	○ 6 months (*n* = 22 vs. 29): 3 (14 %) vs. 5 (17 %)	
		○ 9 months (*n* = 18 vs. 32): 3 (17 %) vs. 5 (16 %)	
		○ 15 months (*n* = 16 vs. 21): 5 (31 %) vs. 5 (24 %)	
		○ 2–5 years (*n* = 9 vs. 17): 2 (22 %) vs. 4 (24 %)	
		▪ Worsened BCVA - 20/70 or worse at:	
		○ 3 months (*n* = 26 vs. 32): 5 (19 %) vs. 4 (12 %)	
		○ 6 months (*n* = 22 vs. 29): 4 (18 %) vs. 6 (21 %)	
		○ 9 months (*n* = 18 vs. 32): 2 (11 %) vs. 8 (25 %)	
		○ 15 months (*n* = 16 vs. 21): 4 (25 %) vs. 5 (24 %)	
		○ 2–5 years (*n* = 9 vs. 17): 3 (33 %) vs. 5 (29 %)	
	- Included were CRVO and hemi-CRVO patients with only background diabetic retinopathy, but those who had active NV, VH, traction detachment, or other complications influencing the VA or fields were excluded		
	- Those with elevated IOP with documented normal visual field before the onset of CRVO were included		
	▪ Baseline comorbidities: arterial hypertension (45 %), ischemic heart disease (29 %), diabetes mellitus (21 %), transient ischemic attack/cerebrovascular accident (3 %)		

Baseline data were not reported for ischemic patients separately

*AMD* age-related macular degeneration, *anti-VEGF* anti-vascular endothelial growth factor, *BCVA* best corrected visual acuity, *CRT* central retinal thickness, *CRVO* central retinal vein occlusion, *CVOS* Central Retinal Vein Occlusion Study, *ETDRS* Early Treatment Diabetic Retinopathy Study, *iCRVO* ischemic central retinal vein occlusion, *IOP* intraocular pressure, *LogMAR* logarithm of the minimum angle of resolution, *LP* light perception, *ME* macular edema, *NV* neovascularization, *NVG* neovascular glaucoma, *OCT* optical coherence tomography, *RAPD* relative afferent pupillary defect, *VA* visual acuity, *VH* vitreous hemorrhage

### Economic outcomes

Key economic data reported across all studies were cost of treatment, administration costs, cost per QALY, and ICER, and key therapies studied were ranibizumab, dexamethasone intravitreal implants, and aflibercept. For two studies conducted in the UK, the analysis was carried out from a UK NHS perspective; [33, 34] in contrast, studies from the USA (*n* = 2) [31, 35], Sweden (*n* = 1) [29], and Canada (*n* = 2) [30, 32] used a payer/healthcare perspective, and another study from Canada used a societal perspective. All included studies calculated the costs of ME secondary to iCRVO but lacked economic data for NV complications. One study expressed costs in 2011 GBP [33], another in 2012 Canadian dollars [30], and another in 2011 USD [31]. For the rest of the studies, which did not report the currency-year, the year of publication was assumed to be the currency-year [29, 32, 34]. Sensitivity analysis was reported in all [29–31, 33–35] but one study [32]. All costs are reported in 2015 values.

In the UK, the ICER of ranibizumab versus observation was £18,381, which included cost of treatment, adverse events, and cost of blindness [33]. In Sweden, aflibercept was dominant, being both less costly (incremental cost of − £2654) and more effective (incremental QALY of 0.061) than ranibizumab [29]. In the USA, the ICER for ranibizumab was £24,882 versus dexamethasone intravitreal implant from a payer perspective [31]. For a patient cohort aged 66–68 years, Haig et al. [32] found that the ICERs for ranibizumab were £16,243 and £1218 (2015 values) if conducted through a Canadian payer perspective and a societal perspective, respectively.

The incremental cost-utility ratios for dexamethasone intravitreal implant versus observation were £12,492 and £8168 as conducted through a Canadian payer perspective and a societal perspective, respectively [30]. This analysis included cost of treatment, cost of adverse events, and cost of blindness [30]. In the USA, the ICER for dexamethasone intravitreal implant was £13,913 versus observation from a payer perspective [31]. The ICER of dexamethasone intravitreal implant versus observation was £17,757, which included only the cost of treatment [34]. In another cost-effectiveness analysis conducted in the USA, the ICER of dexamethasone intravitreal implant compared to observation was reported to be £14,983, which was sensitive to the percentage of patients incurring CRVO in the best-seeing eye, the risk of fellow eye occurrence, and cost of vision loss [35]. Additional details about the included studies are shown in Table 6.

**Table 6:** Study characteristics and economic outcomes data reported in the included studies

Study details	Discounting	Economic endpoints measured	Costs reported	Adjusted costs in 2015 GBP^a^	Sensitivity analyses results
ANTI-VEGF TREATMENTS					
Taylor et al., 2014 [33]	3.50 %	Ranibizumab			At a willingness-to-pay threshold of £30,000/QALY gained, the probability of ranibizumab being cost-effective is 68.3 %
UK; CEA		Cost per treatment	£742.17	£798	
T: ranibizumab		Cost of administration	£192.00	£206	
C: observation		Total costs	£20,646	£22,189	
Cost year: 2011		QALYs	7.383	NA	
		Observation			
		Total costs	£11,430	£12,284	
		QALYs	6.844	NA	
			£17,103	£18,381	
		ICER, cost/QALY	£423	£455	
		Incremental cost per month free from blindness			
Eriksson et al., 2014 [29]	NR	Aflibercept			PSA showed that aflibercept was dominating over ranibizumab in 70 % of the simulations
Sweden; CEA		Incremental costs	−35,000 SEK	−£2654	
T: aflibercept		Incremental QALYs	0.061	NA	
C: ranibizumab		Ranibizumab	−8537 SEK	−£647	
Cost year: not reported^a^		Incremental drug cost	−5793 SEK	−£439	
		Incremental administration cost			
Duff et al., 2012 [31]	3 %	Ranibizumab			PSA demonstrated that at a threshold of $50,000/QALY, ranibizumab was cost-effective in 88.3 % of simulations
USA; CUA		Product cost per vial	$1950	£1419	
T: ranibizumab		Cost of adverse events	$376	£274	
C: dexamethasone intravitreal implants		Dexamethasone	$1295	£942	
Cost year: 2011		Product cost per implant	$180	£131	
		Cost of administration	$63	£46	
		Cost of adverse events			
		ICER, cost/QALY	$34,204	£24,882	
Haig et al., 2012 [32]	5 %	ICER, cost/QALY			Not reported
Canada; CUA		Healthcare perspective	CAD$28,046	£16,243	
T: ranibizumab		Societal perspective	CAD$2103	£1218	
C: observation					
Cost year: not reported					
STEROID TREATMENTS					
Vicente et al., 2013 [30]	5 %	ICUR, cost/QALY			Throughout the 1000 iterations of the PSA the ICER consistently fell below a willingness-to-pay threshold of CAD$50,000/QALY gained. Although robust, the model was most sensitive to age of entry and the utilities used for both the best-seeing eye and worst-seeing eye
Canada; CUA		Public payer perspective	CAD$21,568	£12,492	
T: dexamethasone 700 μg intravitreal implant		Societal perspective	CAD$14,103	£8168	
C: observation					
Cost year: 2012					
Duff et al., 2012 [31]	3 %	Dexamethasone			At low cost-effectiveness thresholds (<$19,000/QALY), steroid treatment was most likely to be cost-effective
USA; CUA		Product cost per implant	$1295	£942	
T: dexamethasone intravitreal implants		Cost of administration	$180	£131	
C: steroids: triamcinolone acetonide		Cost of adverse events	$63	£46	
Cost year: 2011		Steroid	$3	£2	
		Product cost	$123	£89	
		Cost of adverse events	$19,126	£13,913	
		ICER, cost/QALY			
Hayward et al., 2011 [34]	NR	Dexamethsone			PSA showed that at a threshold of £30,000, dexamethasone was a cost-effective option in 85.2 % of simulations
UK; CEA		Total costs	£12,332	£13,254	
T: dexamethasone intravitreal implants		QALYs	11.18	NA	
C: observation		Observation			
Cost year: not reported^a^		Total costs	£7600	£8168	
		QALYs	10.89	NA	
		ICER, cost/QALY	£16,522	£17,757	
Kowalski et al., 2011 [35]	3 %	ICER, cost/QALY	$20,597	£14,983	PSA demonstrated that the ICERs fall below a threshold of $50,000 per QALY in 92 % of simulations. ICER was sensitive to the percentage of patients incurring CRVO in the best-seeing eye, risk of fellow eye occurrence, and cost of vision loss
USA					
T: dexamethasone 700 μg intravitreal implant					
C: observation					
Cost year: not reported^a^					

*C* comparator, *CEA* cost-effectiveness analysis, *CRVO* central retinal vein occlusion, *CUA* cost-utility analysis, *ICER* incremental cost-effectiveness ratio, *NA*, not applicable, *PSA* probabilistic sensitivity analysis, *QALY* quality-adjusted life year, *T* treatment

^a^Year of publication was considered as the cost year for calculation purposes

### Risk of bias

Using the Cochrane risk-of-bias assessment, the types of bias evaluated for clinical studies were: selection bias: patients not assigned to an intervention or control group using random sequence generation (eight studies [20, 21, 23–28]), or the allocation of participants not concealed (seven studies [20, 21, 23–26, 28]); performance bias: lack of blinding of participants and personnel (seven studies [20, 21, 23–26, 28]); detection bias: blinding of investigators was not done as blinding reduces confounding related to the knowledge of intervention assignment (seven studies [20, 21, 23–26, 28]); attrition bias: incomplete outcomes data due to omission of some participants from the reports of analyses (seven studies [17–21, 26, 27],); reporting bias: selective reporting of study measures (six studies [17–19, 21, 26, 27]); and other biases inherent in various study designs (no studies) [15]. A summary of the risk of bias among the included studies are shown in Table 7. Overall, the risk of bias was high.

**Table 7:** Risk of bias in the included studies

Study	Random sequence generation (selection bias)	Allocation concealment (selection bias)	Blinding of participants and personnel (performance bias)	Blinding of outcome assessment (detection bias)	Incomplete outcome data (attrition bias)	Selective reporting (reporting bias)	Other bias
Korobelnik et al. 2014 [17]	+	?	+	+	–	–	+
Brown et al. 2013 [19]	+	?	+	+	–	–	+
Boyer et al. 2012 [18]	+	?	+	?	–	–	+
Wittstrom et al. 2012 [16]	?	?	?	?	+	+	+
Campochiaro et al. 2008 [27]	–	?	+	?	–	–	+
Asano et al. 2007 [20]	–	–	–	–	–	+	+
Ramezani et al. 2006 [21]	–	–	–	–	–	–	+
Jonas et al. 2005 [26]	–	–	–	–	–	–	+
Tabatabaii et al. 2008 [23]	–	–	–	–	+	+	+
Parodi et al. 2007 [22]	+	+	?	?	+	+	+
Feltgen et al. 2007 [24]	–	–	–	–	+	+	+
Mirshahi et al. 2005 [25]	–	–	–	–	+	+	+
Hayreh et al. 2011 [28]	–	–	–	–	+	+	+

– is used to donate high risk, + to denote low risk, and ? to denote an unclear risk

## Discussion

Our systematic review found studies reporting treatments for iCRVO that included anti-VEGFs, steroids, anticoagulants, and procedural treatments. Treatments commonly targeted the complications of ME and NV. Although complications secondary to iCRVO were successfully treated, BCVA failed to improve and patients continued to have severe vision loss or near-blindness. The rate of development of complications during treatment or follow-up was only reported for procedural treatments. There were no data in the studies on the relationship between the various complications of iCRVO. Additionally, there was a lack of economic evidence for iCRVO population. A number of definitions were used for iCVRO, but they mainly used a combination of criteria within the Hayreh and CVOS classifications.

### Treatments for ischemic central retinal vein occlusion

Treatments for iCRVO complication of ME with anti-VEGFs included aflibercept and ranibizumab. Aflibercept treatment improved BCVA in iCRVO patients but population size of iCRVO in the trials was small, and trials with larger sample sizes may be needed for more conclusive results [17–19]. Ranibizumab showed an encouraging improvement in BCVA and also decreased excess foveal thickness in iCRVO patients [27]. However, the numbers of patients were smaller, follow-up was short, and there was a lack of control arm. Thus, these results cannot be considered definitive.

The combination of anti-VEGF bevacizumab injection and PRP resolved anterior-segment NV and prevented an increase in intraocular pressure, but did not lead to an improvement in BCVA in iCRVO patients [16]. Also, bevacizumab caused systemic and ocular adverse events [16]. Aflibercept, ranibizumab, and triamcinolone injections reduced CRT, but the level of significance of this reduction was not reported. Anti-VEGFs reduced ME in iCRVO patients effectively; however, their effect on neovascular complications was not clear. The authors of the rubeosis anti-VEGF (RAVE) trial concluded that anti-VEGFs only delay the neovascular complications in iCRVO and do not treat the underlying blockage of the blood flow in the central retinal vein [36]. Overall, it appears that anti-VEGF treatments provide a short-term impact.

Among various steroids which are available for treating ME [37] (such as triamcinolone acetonide, dexamethasone, and fluocinolone), clinical efficacy of triamcinolone was studied in iCRVO and economic evidence was available for dexamethasone [30, 31, 34, 35] but its clinical efficacy has not been studied recently. Similar to anti-VEGFs, the effects of triamcinolone acetonide on BCVA were sustained only for the short term (less than 6 months) [20, 21, 26].

Our review found that procedural treatments are not successful in improving the vision or even preventing further vision loss in iCRVO. Retinal endovascular lysis, PRP, selective PRP, and photodynamic therapy with verteporfin did not improve BCVA [22, 24]. Moreover, the majority of procedural treatments caused vision to deteriorate. The surgical induction of chorioretinal venous anastomosis may improve BCVA and prevent NV in iCRVO [25], but randomized studies with larger sample sizes are needed. These findings are similar to another review that evaluated the effectiveness of surgical treatments in CRVO patients [38]. In that review, while laser and other surgical interventions were still important treatment modalities, they were mostly reserved for severe cases of ischemia. Hence, lack of visual improvement may have been due to the overall poor prognosis of ischemic eyes requiring surgery. However, the number of post-operative complications were high.

One study used aspirin for its anticoagulant properties [28], but this also did not improve vision. In fact, patients in the aspirin study showed worse vision, more retinal hemorrhages, and more visual-field loss than non-ischemic patients [28]. Aspirin was not recommended in ischemic patients.

At present, therapies used for the acute treatment of CRVO may include medical therapy with anticoagulants, fibrinolytics, corticosteroids, acetazolamide, and isovolemic hemodilution [3], all of which aim to improve venous blood flow in the acute setting. However, such early treatments are generally controversial and off-license, and few patients get detected that early. Even with the use of current therapies, some eyes with iCRVO end up blind and painful and, ultimately, enucleation (removal of the eyeball) may be necessary to provide comfort to patients [39]. Thus, there is a need for curative treatments and better preventative treatments in iCRVO.

### Definitions of ischemia

It is possible that differences in the results of BCVA could arise from the lack of a standardized definition of ischemia. Hayreh et al. differentiated ischemic eyes based on the propensity for neovascular complications using functional tests such as visual acuity, visual fields, RAPD, electroretinography, and two morphologic tests (slit-lamp ophthalmoscopy and fluorescein fundus angiography); [9] whereas CVOS defined iCRVO when there is fluorescein angiographic evidence of more than 10 optic disc areas of capillary non-perfusion [10]. As observed in the literature, few studies used only one of these criteria; indeed, most studies used a mix of these criteria to define ischemia. Also, as pointed out in the interim guidelines published by the Royal College of Ophthalmologists, no evidence of the correct combination of these two leading definitions exists that can best define iCRVO [40]. Since this systematic review was completed, the results of the CRYSTAL study have been published, which looked at the effectiveness of ranibizumab in CRVO [41]. In this study by Larsen et al. a new definition of ischaemia was proposed based on fluorescein angiography macular subfield analysis [41]. This new definition does not conform to the definition used by Hayreh et al. [9], but it is valuable contribution to the field. More work is needed in this area as a need exists to standardize the definition of ischemia that can help disease prognosis and treatment decisions.

### Complications in ischemic central retinal vein occlusion

Ischemia in CRVO leads to complications such as ME, NV, or VH. The relationship between these complications is often under-examined. A retrospective study conducted by Chen et al. found that the incidence of developing NVG in pre-existing glaucoma eyes was significantly higher in groups with ischemia and an intraocular pressure greater than 20 mmHg [42]. It is important to detect such relationships between the prominent complications of iCRVO as this can help change the treatment paradigms and reduce the clinical burden of the disease.

In order to treat the complications of iCRVO, various treatments are employed but these can often lead to their own complications or adverse events. Serious ocular adverse events are observed in anti-VEGF treatments; [18, 19, 27] however, they are not reported separately by ischemic status of the patient. Complications often develop following the procedural treatments, hence surgical options should be selected with caution. In order to treat the complications caused by the treatments, additional therapies or procedures are required [24], which further increases the disease burden.

### Economic outcomes

We did not find any study reporting data on the cost of therapies to prevent or treat complications in the population defined as ischemic, suggesting a major gap in the literature for this population. In lieu of a defined ischemic population, we assumed the presence of NV complications and persistent ME to be an indicator of ischemia in the CRVO population. All economic studies reported cost outcomes in the CRVO population with persistent ME, and no data were found for other complications such as NV or NVG. We also did not identify any publications assessing economic outcomes for bevacizumab, triamcinolone, and procedural treatments, which are often used in iCRVO.

**Fig. 2:**
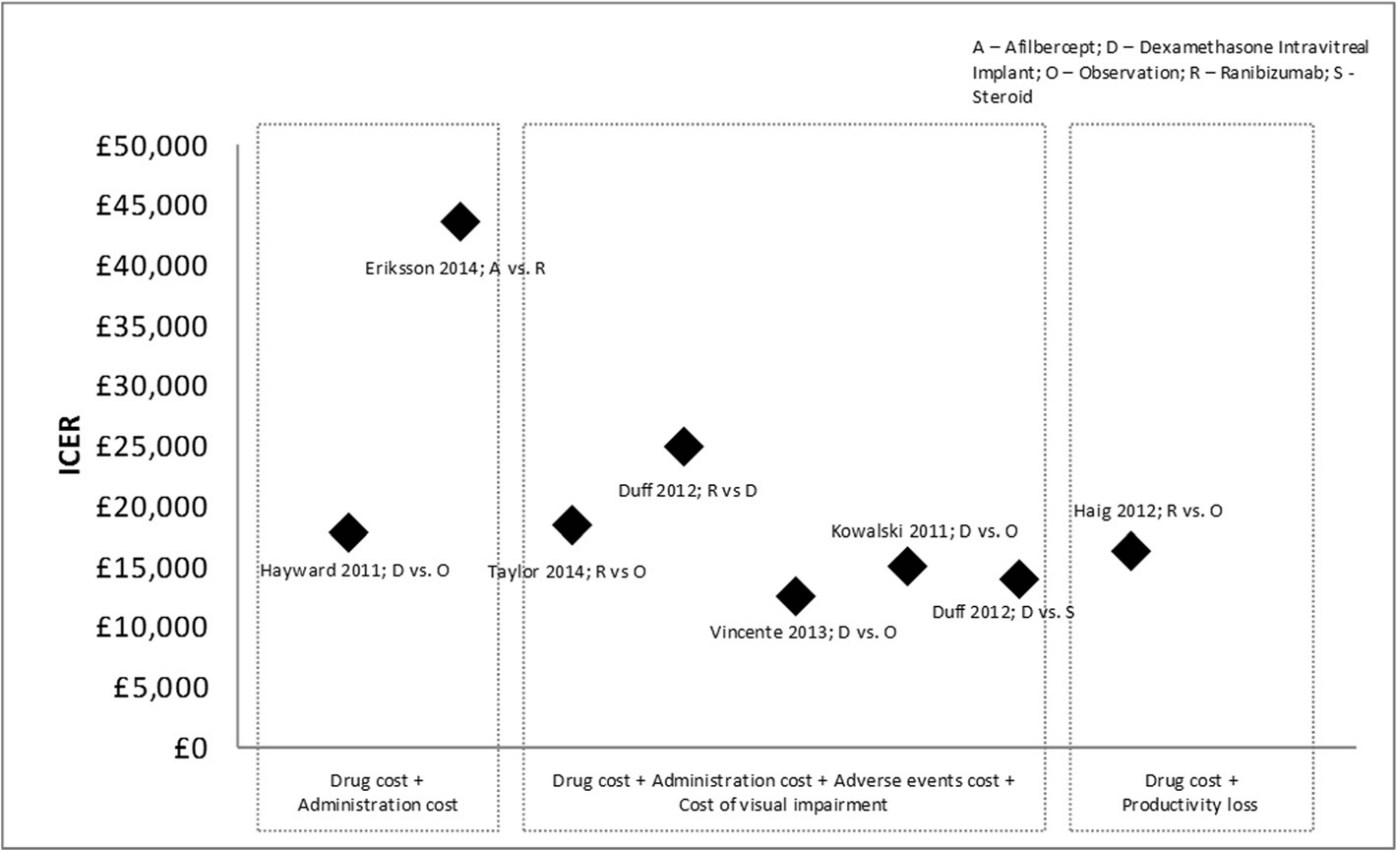
Incremental cost-effectiveness ratio (ICER) values grouped by cost component. Amounts are in 2015 GBP

Cost components included across all analyses also varied to some extent. All economic studies considered only direct costs or components of direct costs. For example, two studies included cost of treatment and its administration in their analysis [29, 31], whereas four included costs associated with adverse events in their analysis [30, 31, 33, 35]. Since most therapies are associated with complications, cost models cannot be considered robust with consideration of cost of these complications and adverse events. Moreover, none of the studies evaluated indirect costs of complications of iCRVO. Since iCRVO can lead to severe vision loss, it can be assumed that the indirect cost burden will be high. Commonly, observation or no treatment was considered as the comparator. We found only two analyses making direct comparison between active treatments. These were for aflibercept versus ranibizumab and for ranibizumab versus dexamethasone intravitreal implants [29, 31]. Figure 2 shows the ICER values reported across studies with monetary findings converted to 2015 GBP values and grouped by cost components considered in the analysis. All but one of the ICER values are below the accepted £30,000/QALY threshold [43]. Although these therapies stay under the ICER threshold, it is important to note that they are not curative treatments and they only ameliorate the symptoms of the disease. However, the low ICER values are a reflection of significant impacts on quality of life and/or QALYs. Thus, further research is needed in this population to further understand both the clinical effects and the quality-of-life aspects.

### Study strengths and limitations

To the best of our knowledge this is the first systematic review to assess clinical outcomes and economic outcomes in iCRVO. Also, it is the only report that presents the various definitions of ischemia and rates of complication development from published studies. A major strength of this research is the comprehensive, structured, and systematic approach undertaken to search the literature and conference proceedings to identify all studies that report clinical and economic outcomes in the iCRVO segment. Moreover, BCVA, which is reported in the literature with various units, was converted to a single unit of LogMAR. This homogenizes the results for easier understanding. It should be noted that there are considerable methodological limitations in the included studies. While evaluating clinical outcomes, except for two studies [16, 22], all treatments are compared to sham injection, no treatment, pre-treatment, or the same treatment but with a different dose. Thus, there is a lack of head-to-head trials demonstrating the relative efficacy of treatments. Although meta-analyses exist for CRVO [44] there are no meta-analyses comparing various treatments for different complications in the iCRVO population. Among the included economic studies, two studies made direct comparisons between active treatments, but various other treatments often used to iCRVO complications were not studied.

Additionally, there was a lack of RCTs with long follow-up durations and the ischemic population was poorly represented in bigger trials. Only a few trials included in this review had a follow-up of more than 12 months [17, 19, 25, 28]. A trial conducted in iCRVO patients found that the complication of edema reoccurred after the discontinuation of ranibizumab. When ranibizumab injections were withheld for 3 months, about half of patients had recurrent edema along with the loss of visual acuity gains through the treatment [36]. Trials with longer follow-up can provide long-term patient outcomes which may be helpful in understanding the treatment. Furthermore, the proportion of patients with ischemia is dramatically smaller than that of non-ischemic patients in trials concerning the CRVO population. Even when a trial recruits only iCRVO patients, the sample size is very small. Thus, there are no trials with large numbers of ischemic patients, leading to uncertainties in the robustness of the evidence for this group of patients. The majority of cost evidence was obtained from conference proceedings, which leads to limited understanding of the economic aspect of iCRVO. It was difficult to compare studies on key cost drivers in order to understand the differences because of the lack of detail being reported.

A few limitations should be considered when interpreting these findings. BCVA and CRT were presented at various time points, and this varied between studies, it was difficult to make a direct comparison. It was not possible to convert the change in BCVA into LogMAR units for two studies [17, 18] as the baseline BCVA data were not available. Any indirect comparisons must be made with extreme caution as the patient population, complications secondary to CRVO, follow-up period, treatments, economic analysis perspective, and countries differ from study to study.

Researchers can expand the review findings by adding the results from retrospective case series and individual case studies, which comprises the majority of literature on iCRVO. Combination therapies can be explored in iCRVO, which may have the potential to improve vision and reduce complications. A trial in our review shows the benefits of bevacizumab injection in combination with PRP [16], while another trial highlights the avoidance of PRP in all iCRVO patients by choosing selective PRP [22]. A combination of selective PRP and bevacizumab injections may be an effective strategy in iCRVO patients suffering from anterior-segment NV. Extensive research is still needed on the role of anti-VEGFs in treating the complications of iCRVO. Researchers can add to economic evidence of iCRVO by conducting cost analyses specific to iCRVO patient population. The therapeutic care of iCRVO awaits an innovative therapy that can improve the blood flow to the center of the retina.

## Conclusions

In conclusion, there is no high-level evidence for any current intervention being effective in a population of exclusively iCRVO cases. Furthermore, there is no solid evidence that anti-VEGF treatment, which is highly effective in CRVO without ischemia, does anything to prevent neovascularization in iCRVO. According to published studies, existing treatments reduce only the complications of iCRVO and do not significantly improve vision impairment, or do so only temporarily. Notwithstanding the scarcity of studies, there is a pressing need for innovative curative and preventive treatments in iCRVO as none of the current treatments solve the significant clinical and economic burden of this blinding condition.

## Abbreviations

AAO, American Academy of Ophthalmology; BCVA, best-corrected visual acuity; CDSR, Cochrane Database of Systematic Reviews; CENTRAL, Cochrane Central Register of Controlled Trials; CRT, Central Retinal Thickness/Macular Thickness; CRVO, central retinal vein occlusion; DARE, Database of Abstracts of Reviews of Effects; ERIC Education Resources Information Center; HTA, Health Technology Assessment; ICER, incremental cost-effectiveness ratio; iCRVO, ischemic central retinal vein occlusion; ISPOR, International Society of Pharmacoeconomics and Outcomes Research; LogMAR, logarithm of the minimum angle of resolution; ME, macular edema; MeSH, Medical Subject Headings; NHS EED UK, National Health Service Economic Evaluation Database; NV, neovascularisation; NVG, neovascular glaucoma; PRP, pan-retinal photocoagulation; QALY, quality-adjusted life year; RAPD, relative afferent pupillary defect; RAVE, rubeosis anti-vegf trial; RCT, randomised-controlled trial; VH, vitreous hemorrhage
